# Perioperative exercise capacity in chronic liver injury patients with hepatocellular carcinoma undergoing hepatectomy

**DOI:** 10.1371/journal.pone.0221079

**Published:** 2019-08-14

**Authors:** Masaki Kaibori, Kosuke Matsui, Kengo Yoshii, Morihiko Ishizaki, Junji Iwasaka, Takumi Miyauchi, Yutaka Kimura

**Affiliations:** 1 Department of Surgery, Hirakata Hospital, Kansai Medical University, Hirakata, Osaka, Japan; 2 Department of Mathematics and Statistics in Medical Sciences, Kyoto Prefectural University of Medicine, Kyoto, Japan; 3 Health Science Center, Hirakata Hospital, Kansai Medical University, Hirakata, Osaka, Japan; Boston University, UNITED STATES

## Abstract

Dynamic assessment of preoperative exercise capacity may be a useful predictor of postoperative prognosis. We aimed to clarify whether perioperative exercise capacity was related to long-term survival in hepatocellular carcinoma patients with chronic liver injury undergoing hepatectomy. One hundred-six patients with hepatocellular carcinoma underwent pre- and postoperative cardiopulmonary exercise testing to determine their anaerobic threshold, defined as the point between carbon dioxide production and oxygen consumption per unit of time. Testing involved 35 items including blood biochemistry analysis, in-vivo component analysis, dual-energy X-ray absorptiometry, and cardiopulmonary exercise testing preoperatively and 6 months postoperatively. We classified patients with anaerobic threshold ≥ 90% 6 months postoperatively compared with the preoperative level as the maintenance group (n = 78) and patients with anaerobic threshold < 90% as the decrease group (n = 28). Five-year recurrence-free survival rates were 39.9% vs. 9.9% (maintenance vs. decrease group) (hazard ratio: 1.87 [95% confidence interval: 1.12–3.13]; P = 0.018). Five-year overall survival rates were maintenance: 81.9%, and decrease: 61.7% (hazard ratio: 2.95 [95% confidence interval: 1.37–6.33]; P = 0.006). Multivariable Cox proportional hazards models showed that perioperative maintenance of anaerobic threshold was an independent prognostic indicator for both recurrence-free- and overall survival. Although the mean anaerobic threshold from preoperative to postoperative month 6 decreased in the exercise-not-implemented group, the exercise-implemented group experienced increased anaerobic threshold, on average, at postoperative month 6. The significant prognostic factor affecting postoperative survival for chronic liver injury patients with HCC undergoing hepatectomy was maintenance of anaerobic threshold up to 6 months postoperatively.

## Introduction

Major surgery increases oxygen demand by approximately 40%, which may place severe stress on cardiopulmonary reserve [1]. Patients with high cardiopulmonary risk have traditionally been assessed using tests such as transthoracic echocardiography, dobutamine stress echocardiography, radionuclide ventriculography, and spirometry. However, these assessments have not been validated as preoperative screening tests, and provide primarily static measurements of cardiopulmonary performance [2–4]. Walking distance and ability to climb stairs are subjective measurements of exercise tolerance, and have been shown to predict perioperative complications [5,6]. However, these measurements lack objectivity and do not detect silent cardiopulmonary abnormalities. Dynamic assessment of preoperative exercise capacity may be a useful predictor of short- and long-term postoperative prognosis. Cardiopulmonary exercise (CPX) testing measures oxygen uptake at increasing levels of work and predicts cardiopulmonary performance under stress, such as after surgery. In older patients undergoing major abdominal surgical procedures, the majority of deaths from cardiopulmonary complications occur in patients with an anaerobic threshold (AT) < 11 ml/min/kg [7,8].

Hepatocellular carcinoma (HCC) is the fifth-most-common cancer worldwide [9]. Maintenance of good perioperative nutrition and metabolism may improve the prognosis of patients with HCC undergoing hepatectomy [10, 11]. To date, few studies have examined the usefulness of pre- and postoperative CPX testing in patients undergoing hepatectomy. In the present study, we aimed to clarify whether perioperative exercise capacity was related to long-term survival in HCC patients with chronic liver injury undergoing hepatectomy.

## Materials and methods

### Patients

HCC patients with chronic hepatitis or cirrhosis who were scheduled for liver resection at Hirakata Hospital of Kansai Medical University (Osaka, Japan) between March 2010 and September 2015 were screened for inclusion in this study. A total of 111 patients with HCC underwent curative resection (defined as macroscopic removal of all tumor). No in-patient mortality occurred, and we analyzed data for 106/111 patients. We excluded data for the remaining five patients because these patients were followed at other hospitals. All patients gave written informed consent to participate in this study, and the study protocol was approved by the institutional ethics committee of Kansai Medical University (reference number: KMU 2018082).

### Cardiopulmonary exercise testing

Patients underwent preoperative CPX testing using a bicycle ergometer with an incremental protocol (5.0, 7.5, and 10 W/min). Twelve-lead electrocardiography was used to monitor heart rate, ST segment deviation, and arrhythmias, at rest and continuously during exercise and recovery periods. Blood pressure was recorded at rest and every 2 min during exercise and recovery periods. Peak oxygen consumption per unit of time (VO_2_) was obtained from breath-by-breath analysis of expired air. Peak VO_2_ was defined as the highest mean value during exercise when the subject could no longer continue pedaling at 60 rpm. AT, indicating the onset of metabolic acidosis, was defined as the break point between carbon dioxide production and VO_2_ [12], or the point at which the ventilatory equivalent for oxygen and end-tidal oxygen partial pressure curves reached their respective nadirs before beginning to increase again [13]. Thus, AT was set at the time of maximum fat combustion [14]. The respiratory compensation point was set at the point at which the ventilatory equivalent for carbon dioxide was lowest before a systemic increase, and when the end-tidal carbon dioxide partial pressure reached a maximum and began to decrease [15]. Exercise was stopped when the patient requested because of fatigue, pain, or headache, or if there was failure to maintain a speed greater than 40 rpm for more than 30 seconds despite encouragement.

For included patients wishing to begin exercise therapy, the following protocol was performed perioperatively, tailored to each patient: [16] Exercise was started as soon as possible after diagnosis, up to 1 month preoperatively, and was resumed from 1 week postoperatively, and continued for 6 months (> 3 times a week). The program consisted of three 60-minute exercise sessions per week, and each session included 5 minutes of stretching exercises, 30 minutes of walking at an intensity based on the AT of each patient, 20 minutes of targeted stretching exercises, and 5 minutes of cooling down with stretching. Once or twice a month postoperatively, a medical doctor and exercise trainer confirmed the frequency and quantity of exercise each patient undertook. Fifty-nine patients (exercise-implemented group) engaged in exercise therapy perioperatively and 47 did not (exercise not-implemented group).

CPX measurements were performed preoperatively and 6 months postoperatively to assess postoperative changes. The median (interquartile range) 6-month postoperative AT VO_2_ level was 101.5% (89.3%–113.7%). Patients were classified based on whether they had an AT VO_2_ level of 90% (25th percentile of the AT VO_2_ level) 6 months postoperatively compared with their preoperative level. Patients who had an AT VO_2_ level ≥ 90% 6 months postoperatively compared with their preoperative level were classified as the maintenance group (n = 78), and patients with AT VO_2_ < 90% for the same comparison were classified as the decrease group (n = 28).

### Clinical variables and surgery

Before surgery, each patient underwent peripheral blood count, general biochemical blood laboratory testing, conventional liver function testing, and measurement of indocyanine green retention rate at 15 min. Hepatitis virus infection screening was performed by testing for hepatitis B surface antigen and hepatitis C virus antibody (HCVAb). Alpha-fetoprotein and protein induced by vitamin K absence/antagonism-II (PIVKA-II) levels were measured in all patients. We used two methods to determine body composition: dual-energy X-ray absorptiometry (DEXA) [17] and bioelectrical impedance analysis (BIA) [18] preoperatively and 6 months postoperatively. Total weight, nonfat content, and fat content were measured by whole-body DEXA. BIA was performed using the whole-body eight-electrode approach with a 5–500 kHz multifrequency impedance analyzer (InBody720, Biospace Co., Ltd, Tokyo, Japan). Skeletal muscle content, fat content, and body mass index were also measured.

Surgical procedures were classified according to the Brisbane terminology proposed by Strasberg et al. [19]. Anatomical resection was defined as resection of the tumor together with the related portal vein branches and corresponding hepatic territory, and was classified as hemihepatectomy (resection of half of the liver), extended hemihepatectomy (right trisectionectomy, or similar procedures on the left or for smaller resections), sectionectomy (resection of two Couinaud subsegments [20]), or segmentectomy (resection of one Couinaud subsegment). All other procedures were classified as nonanatomical resection, which was frequently performed for peripheral or central tumors. Peripheral tumors and those with extrahepatic growth were treated by partial hepatectomy because this procedure achieved sufficient surgical margins. Central tumors located near the hepatic hilum or major vessels were treated by enucleation only because it was too difficult and/or dangerous to remove enough liver tissue to obtain adequate margins. One consultant pathologist reviewed all specimens for histological confirmation of the diagnosis. The width of the surgical margin was measured as the distance from the tumor edge to the resection line.

### Follow-up

Peri- and postoperative complications and deaths were recorded to determine morbidity and mortality following hepatectomy. All surviving patients were followed at least every 3 months after discharge. Follow-up included physical examination, liver function testing, chest radiographs to check for pulmonary metastases, and ultrasonography, computed tomography, or magnetic resonance imaging to check for intrahepatic recurrence. Chest computed tomography was performed if the chest radiograph showed any abnormalities, and bone metastases were diagnosed by bone scintigraphy.

When HCC recurrence was detected by changes in tumor markers or on imaging, recurrence limited to the remnant liver was treated by transarterial chemoembolization, lipiodolization, re-resection, or percutaneous local ablative therapy such as radiofrequency ablation. When extrahepatic metastases were detected, active treatment and/or molecular target drugs such as sorafenib was prescribed in patients with good hepatic functional reserve (Child–Pugh class A or B) and good performance status (0 or 1), while other patients received radiation therapy, alone, to relieve symptoms of bone metastases. Surgical resection was performed in patients with a solitary extrahepatic metastasis and no intrahepatic recurrence.

### Statistical analysis

We compared patients’ clinical characteristics in the two groups using either Wilcoxon’s rank-sum test, chi-square test, or Fisher’s exact test. Probabilities for recurrence-free survival (RFS) and overall survival (OS) by treatment type were calculated by the Kaplan–Meier method. Hazard ratios for RFS and OS and their 95% confidence intervals (CI) were estimated using the univariate Cox model. Multivariate analysis was performed using the Cox proportional hazards model. The following variables were examined as potential prognostic predictors: age, sex, diabetes mellitus, HCVAb status, serum albumin, alanine aminotransferase, total bilirubin, prothrombin activity, platelet count, alfa-fetoprotein and PIVKA-II concentrations, tumor number, maximum tumor size, portal vein and or hepatic vein invasion, tumor stage, volume of hepatectomy, operative blood loss, and the rate of change from preoperative to 6-months-postoperative values including total weight and nonfat content by DEXA, and peak VO_2_ and AT VO_2_ by CPX. Differences between groups in perioperative changes in AT VO_2_ were assessed by two-way repeated measures ANOVA (analysis of variance) and Student's t-test post hoc test. A *P*-value <0.05 (two-sided) was considered statistically significant. All statistical analyses were performed with R version 3.3.1 (R Foundation for Statistical Computing, Vienna, Austria) with the survival and matching packages.

## Results

Table 1 summarizes the preoperative characteristics of both groups. Patients' age and number differed significantly between the two perioperative exercise groups. No difference was detected between groups regarding sex, American Society of Anesthesiologists physical status classification, alcohol intake, diabetes mellitus, hypertension, esophageal and/or gastric varices, hepatitis B surface antigen, HCVAb, Child–Pugh class, indocyanine green retention rate at 15 min, peripheral blood count, general biochemical blood laboratory testing, conventional liver function testing, or serum alfa-fetoprotein and PIVKA-II concentrations.

**Table 1:** Preoperative characteristics of HCC patients classified according to AT VO_2_ change.

Variables	Maintenance (n = 78)		Decrease (n = 28)		*P*
Age, yr	70	(63.0, 73.0)	73	(67.8, 75.0)	**0.023**
Sex					0.604
Male	61	(78%)	20	(71%)	
Female	17	(22%)	8	(29%)	
ASA-PS					0.227
1	8	(10%)	0	(0%)	
2	65	(83%)	26	(93%)	
3	5	(6%)	2	(7%)	
Alcohol intake					0.112
None	53	(68%)	14	(50%)	
Positive	25	(32%)	14	(50%)	
Exercise					**0.004**
Implemented	50	(64%)	9	(32%)	
Not-implemented	28	(36%)	19	(68%)	
Diabetes					0.805
Absent	58	(74%)	20	(71%)	
Present	20	(26%)	8	(29%)	
Hypertension					0.260
Absent	50	(64%)	14	(50%)	
Present	28	(36%)	14	(50%)	
Esophageal and/or gastric varices					1.000
Absent	74	(95%)	27	(96%)	
Present	4	(5%)	1	(4%)	
HBsAg					0.389
Negative	63	(81%)	25	(89%)	
Positive	15	(19%)	3	(11%)	
HCVAb					0.825
Negative	45	(58%)	15	(54%)	
Positive	33	(42%)	13	(46%)	
Child–Pugh class					1.000
A	76	(97%)	27	(96%)	
B	2	(3%)	1	(4%)	
ICGR15, %	13.2	(9.7, 19.0)	12.5	(9.7, 20.1)	0.848
WBC, 10^2^/μL	50.5	(41.0, 60.0)	50.5	(41.0, 64.3)	0.739
Hemoglobin, g/dL	13.1	(12.4, 14.2)	12.7	(11.5, 14.0)	0.060
Hematocrit, %	37.2	(35.4, 39.9)	37.2	(34.4, 40.1)	0.571
Platelet count, ×10^4^/mm^3^	14.6	(11.5, 18.7)	16.5	(13.3, 19.4)	0.337
Albumin, g/dL	4.1	(3.8, 4.3)	3.9	(3.7, 4.3)	0.371
AST, U/L	35.5	(23.5, 50.8)	38.0	(28.5, 66.5)	0.488
ALT, U/L	35.0	(23.0, 53.8)	38.0	(20.5, 59.5)	0.761
Prothrombin activity, %	88.6	(81.7, 96.1)	84.0	(76.0, 92.7)	0.121
ALP, U/L	248.0	(187.8, 346.0)	298.0	(256.8, 347.5)	0.050
γGTP, U/L	58.5	(32.8, 104.3)	48.0	(31.0, 88.0)	0.495
Cholinesterase, U/L	254.0	(197.0, 275.0)	221.0	(175.0, 287.5)	0.231
Total bilirubin, mg/dL	0.8	(0.6, 0.9)	0.6	(0.5, 0.9)	0.252
Creatinine, mg/dL	0.8	(0.6, 0.9)	0.8	(0.7, 1.0)	0.099
CRP, mg/dL	0.1	(0.0, 0.2)	0.1	(0.0, 0.2)	0.830
Triglyceride, mg/dL	104.0	(75.5, 133.0)	91.0	(72.0, 120.0)	0.241
Total cholesterol, mg/dL	163.5	(141.3, 179.0)	171.0	(141.0, 190.0)	0.467
Glucose, mg/dL	106.0	(94.0, 140.3)	102.0	(94.0, 131.0)	0.685
Insulin, μIU/mL	12.6	(6.3, 19.2)	13.0	(8.3, 26.8)	0.520
HbA1c	5.6	(5.3, 6.7)	5.5	(5.0, 5.8)	0.095
Alpha-fetoprotein, ng/mL	7.2	(3.7, 15.6)	11.0	(4.7, 64.4)	0.313
PIVKA-II	39.5	(18.0, 151.0)	109.5	(22.5, 1246.3)	0.126

Data are shown as median (5th percentile, 95th percentile) or *n* (%).

*AT*, anaerobic threshold; *VO*_*2*,_ oxygen consumption; *ASA-PS*, The American Society of Anesthesiologists physical status; *HBsAg*, hepatitis B surface antigen; *HCVAb*, hepatitis C virus antibody; *ICGR15*, indocyanine green retention rate at 15 min; *WBC*, white blood cell count; *AST*, aspartate aminotransferase; *ALT*, alanine aminotransferase; *ALP*, alkaline phosphatase; *γGTP*, γ-glutamyltranspeptidase; *CRP*, C-reactive protein; HbA1c, glycated hemoglobin; *PIVKA-II*, protein induced by vitamin K absence-II.

Preoperative patient characteristics evaluating body composition, and the related indices of CPX including AT VO_2_, AT watt, peak heart rate, and peak VO_2_ were significantly higher in the maintenance group (Table 2). In contrast, total body weight, muscle content, and fat content measured by both BIA and DEXA did not differ between the two groups.

**Table 2:** Preoperative body composition characteristics of HCC patients classified according to AT VO_2_ change.

Variables	Maintenance (n = 78)		Decrease (n = 28)		*P*
Total weight by BIA (rate of change)	97.1	(94.8, 100.4)	98.8	(95.1, 103.0)	0.425
Skeletal muscle content by BIA	99.6	(98.1, 101.2)	100.3	(97.8, 101.9)	0.464
Fat content by BIA	93.6	(82.4, 103.3)	97.2	(84.5, 103.5)	0.910
BMI by BIA	97.3	(94.8, 100.4)	98.9	(95.1, 103.7)	0.422
Total weight by DEXA	97.8	(95.5, 100.1)	98.5	(96.0, 101.3)	0.437
Fat content by DEXA	90.4	(82.6, 102.2)	99.9	(88.8, 102.7)	0.184
Non-fat content by DEXA	100.7	(97.9, 103.3)	99.4	(97.4, 102.3)	0.262
AT VO_2_	105.8	(98.5, 117.1)	81.2	(76.5, 87.8)	**<0.001**
AT W	109.4	(98.4, 126.3)	92.9	(78.8, 104.4)	**<0.001**
AT VE.VCO_2_	97.4	(92.5, 103.2)	104.9	(99.2, 108.8)	**<0.001**
Peak HR	103.5	(96.2, 110.1)	97.5	(88.3, 104.0)	**0.014**
Peak VO_2_	110.0	(96.8, 122.0)	97.4	(84.6, 103.1)	**<0.001**

Data are shown as median (5th percentile, 95th percentile).

*AT*, anaerobic threshold; *VO*_*2*,_ oxygen consumption; *BMI*, body mass index; *BIA*, bioelectrical impedance analysis; *DEXA*, dual-energy X-ray absorptiometry; *W*, Watt; *VE*, minute ventilation; *VCO*_*2*_, carbon dioxide output; *HR*, heart rate.

No difference was detected between the two groups for operative, pathological features, and postoperative characteristics (Table 3). Complications attributable to surgery were noted in eight (10%) patients in the maintenance group and two (7%) patients in the decrease group.

**Table 3:** Operative and postoperative characteristics of HCC patients classified according to AT VO_2_ change.

Variables	Maintenance (n = 78)		Decrease (n = 28)		*P*
Tumor number					0.266
1	54	(69%)	23	(82%)	
2	16	(21%)	2	(7%)	
≥3	8	(10%)	3	(11%)	
Tumor size, cm	3.0	(2.0, 4.0)	3.1	(2.2, 5.3)	0.680
Degree of differentiation					0.217
Well	20	(26%)	11	(39%)	
Moderately	47	(60%)	16	(57%)	
Poorly	0	(0%)	0	(0%)	
Necrosis or unknown	11	(14%)	1	(4%)	
fc					0.810
Absent	22	(28%)	7	(25%)	
Present	56	(72%)	21	(75%)	
vp and/or vv					0.821
Negative	28	(36%)	11	(39%)	
Positive	50	(64%)	17	(61%)	
sm					0.377
Negative	74	(95%)	25	(89%)	
Positive	4	(5%)	3	(11%)	
Underlying liver disease					0.778
Chronic hepatitis	41	(53%)	17	(61%)	
Cirrhosis	27	(35%)	8	(29%)	
Normal	10	(13%)	3	(11%)	
Tumor stage					0.484
I	11	(14%)	6	(21%)	
II	37	(47%)	9	(32%)	
III	24	(31%)	10	(36%)	
IVa	6	(8%)	3	(11%)	
Operative procedure					0.250
Anatomical resection	53	(68%)	15	(54%)	
Non-anatomical resection	25	(32%)	13	(46%)	
Amount of hepatic resection					0.810
Less than hemihepatectomy	56	(72%)	21	(75%)	
More than hemihepatectomy	22	(28%)	7	(25%)	
Operating time, min	353	(270, 408)	305	(230, 416)	0.162
Operative blood loss, ml	712	(378, 1137)	569	(265, 1061)	0.396
Postoperative hospital stay, days	12	(11, 15)	12	(10, 15)	0.484
Blood transfusion					0.741
Absent	69	(88%)	24	(86%)	
Present	9	(12%)	4	(14%)	
Complications					1.000
Absent	70	(90%)	26	(93%)	
Present	8	(10%)	2	(7%)	
Mortality					-
Absent	78	(100%)	28	(100%)	
Present	0	(0%)	0	(0%)	

Data are shown as median (5th percentile, 95th percentile) or *n* (%).

*AT*, anaerobic threshold; *VO*_*2*,_ oxygen consumption; *fc*, capsule formation; *vp*, portal vein invasion; *vv*, hepatic vein invasion; *sm*, surgical margin.

**Fig 1:**
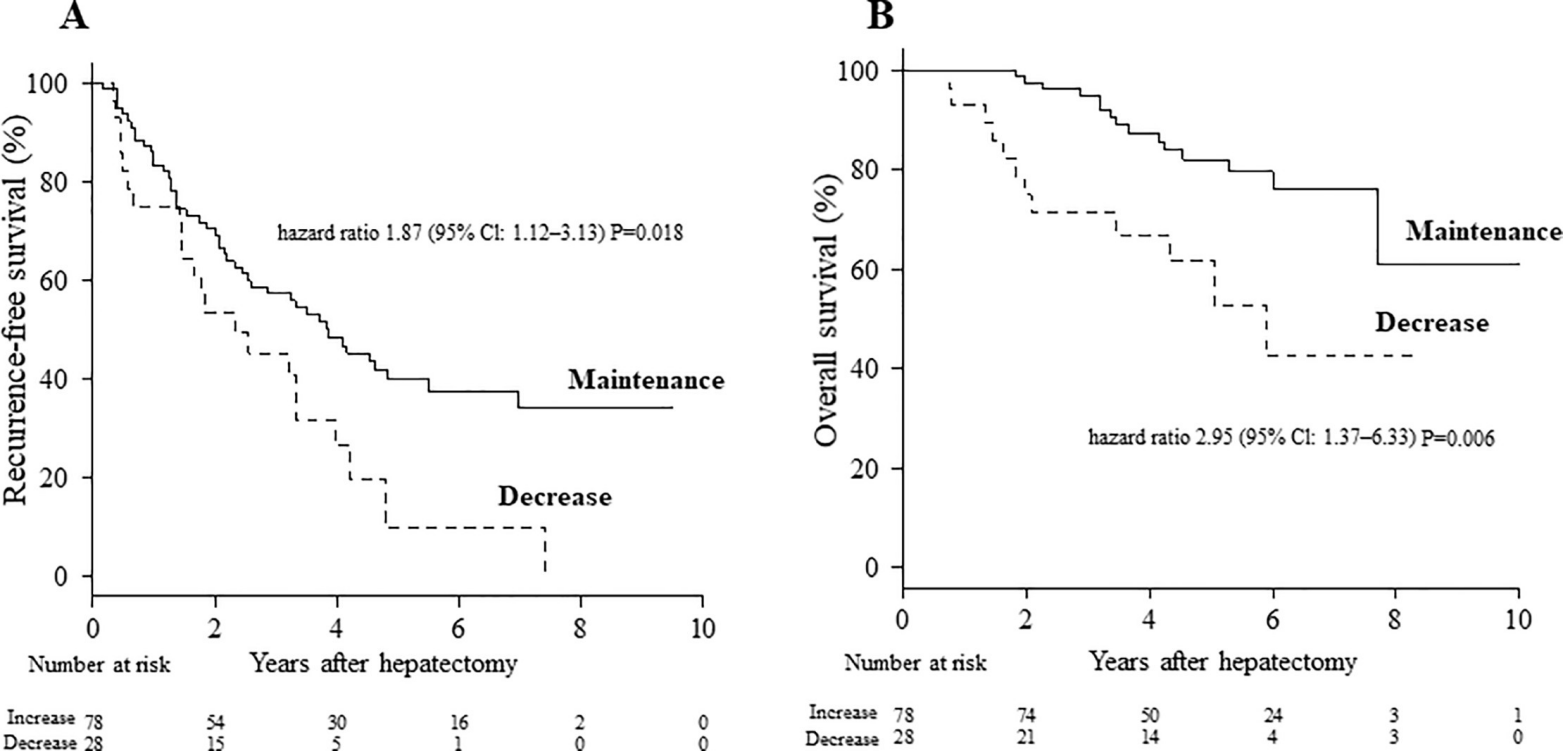
Survival outcomes between the maintenance and decrease groups. A, Recurrence-free survival. B, Overall survival. CI: confidence interval.

Fig 1 shows a comparison of the long-term outcomes between the two groups. The median follow-up period was 57.0 months in the maintenance group and 46.3 months in the decrease group. The 5-year RFS rates were 39.9% for the maintenance group and 9.9% for the decrease group (hazard ratio: 1.87 [95% CI: 1.12–3.13]; *P* = 0.018; Fig 1A). The 5-year OS rates were 81.9% for the maintenance group and 61.7% for the decrease group (hazard ratio: 2.95 [95% CI: 1.37–6.33]; *P* = 0.006; Fig 1B).

Table 4 shows results of a multivariate statistical model incorporating several covariates. Multivariable Cox proportional hazards model identified six independent prognostic predictors for RFS: HCVAb-positive status (hazard ratio, 2.43; 95% CI: 1.19–4.97; *P* = 0.015), prothrombin activity ≥ 87% (hazard ratio, 2.78; 95% CI: 1.31–5.88; *P* = 0.008), PIVKA-II ≥ 46 mAU/mL (hazard ratio, 3.10; 95% CI: 1.36–7.09; *P* = 0.007), tumor number ≥ 2 (hazard ratio, 2.61; 95% CI: 1.13–6.01; *P* = 0.024), peak VO_2_ (rate of change) ≥ 90% (hazard ratio, 2.47; 95% CI: 1.03–5.94; *P* = 0.043), and AT VO_2_ (rate of change) ≥ 90% (hazard ratio, 2.58; 95% CI: 1.22–5.46; *P* = 0.013). Cox models also identified one independent prognostic predictor for OS: AT VO_2_ (rate of change) ≥ 90% (hazard ratio, 5.03; 95% CI: 1.40–18.02; *P* = 0.013; Table 4).

**Table 4:** Hazard ratios for recurrence-free survival and overall survival in HCC patients who underwent hepatic resection: Multivariable cox regression analysis.

Variable	RFS			OS		
	HR	(95% CI)	*P*	HR	(95% CI)	*P*
Age ≥ 70 years (vs. < 70 years)	1.26	(0.63–2.49)	0.514	2.31	(0.73–7.32)	0.156
Sex female (vs. male)	1.89	(0.81–4.43)	0.143	0.59	(0.12–2.95)	0.517
DM present (vs. absent)	0.71	(0.33–1.53)	0.383	2.40	(0.66–8.75)	0.186
HCVAb positive (vs. negative)	2.43	(1.19–4.97)	**0.015**	0.67	(0.19–2.35)	0.530
Platelet count ≥ 15.7×10^4^/mm^3^ (vs. < 15.7×10^4^/mm^3^)	1.47	(0.77–2.80)	0.238	0.69	(0.24–1.96)	0.480
Prothrombin activity ≥ 87% (vs. < 87%)	2.78	(1.31–5.88)	**0.008**	1.46	(0.39–5.46)	0.571
Serum albumin ≥ 4.0 g/dL (vs. < 4.0 g/dL)	0.74	(0.35–1.53)	0.416	0.58	(0.18–1.88)	0.360
ALT ≥ 40 (vs. < 40)	1.44	(0.74–2.82)	0.285	0.71	(0.21–2.46)	0.592
Serum total bilirubin ≥ 0.7 mg/dL (vs. < 0.7 mg/dL)	1.50	(0.66–3.42)	0.338	1.49	(0.32–6.84)	0.610
Alpha-fetoprotein ≥ 8.0 ng/mL (vs. < 8.0 ng/mL)	0.67	(0.34–1.32)	0.248	1.88	(0.48–7.32)	0.361
PIVKA-II ≥ 46 mAU/mL (vs. < 46 mAU/mL)	3.10	(1.36–7.09)	**0.007**	3.37	(0.73–15.64)	0.120
Tumor number ≥ 2 (vs. single)	2.61	(1.13–6.01)	**0.024**	1.78	(0.47–6.71)	0.394
Tumor size ≥ 30 mm (vs. < 30 mm)	0.71	(0.35–1.47)	0.358	0.55	(0.18–1.76)	0.316
vp and/or vv positive (vs. negative)	0.84	(0.33–2.15)	0.723	0.53	(0.12–2.31)	0.395
Tumor stage III or IVa (vs. I or II)	1.55	(0.60–3.99)	0.366	0.56	(0.12–2.60)	0.461
More than hemihepatectomy (vs. less than hemihepatectomy)	0.87	(0.36–2.10)	0.761	0.33	(0.05–2.27)	0.260
Operative blood loss ≥ 500 ml (vs. < 500 ml)	1.20	(0.61–2.38)	0.601	1.62	(0.51–5.20)	0.413
Total weight by DEXA (rate of change) ≥ 100% (vs. < 100%)	0.76	(0.32–1.79)	0.530	0.25	(0.05–1.15)	0.074
Non-fat content by DEXA (rate of change) ≥ 100% (vs. < 100%)	1.42	(0.74–2.75)	0.291	2.65	(0.93–7.58)	0.068
Peak VO_2_ (rate of change) ≥ 90% (vs. < 90%)	2.47	(1.03–5.94)	**0.043**	1.36	(0.31–6.02)	0.687
AT VO_2_ (rate of change) ≥ 90% (vs. < 90%)	2.58	(1.22–5.46)	**0.013**	5.03	(1.40–18.02)	**0.013**

*RFS*, recurrence-free survival; *OS*, overall survival; *HR*, hazard ratio; *CI*, *confidence interval*; *DM*, diabetes mellitus; *HCVAb*, hepatitis C virus antibody; *ALT*, alanine aminotransferase; *PIVKA-II*, protein induced by vitamin K absence-II; *vp*, portal vein invasion; *vv*, hepatic vein invasion; *DEXA*, dual-energy X-ray absorptiometry; *AT*, anaerobic threshold; *VO*_*2*,_ oxygen consumption.

**Fig 2:**
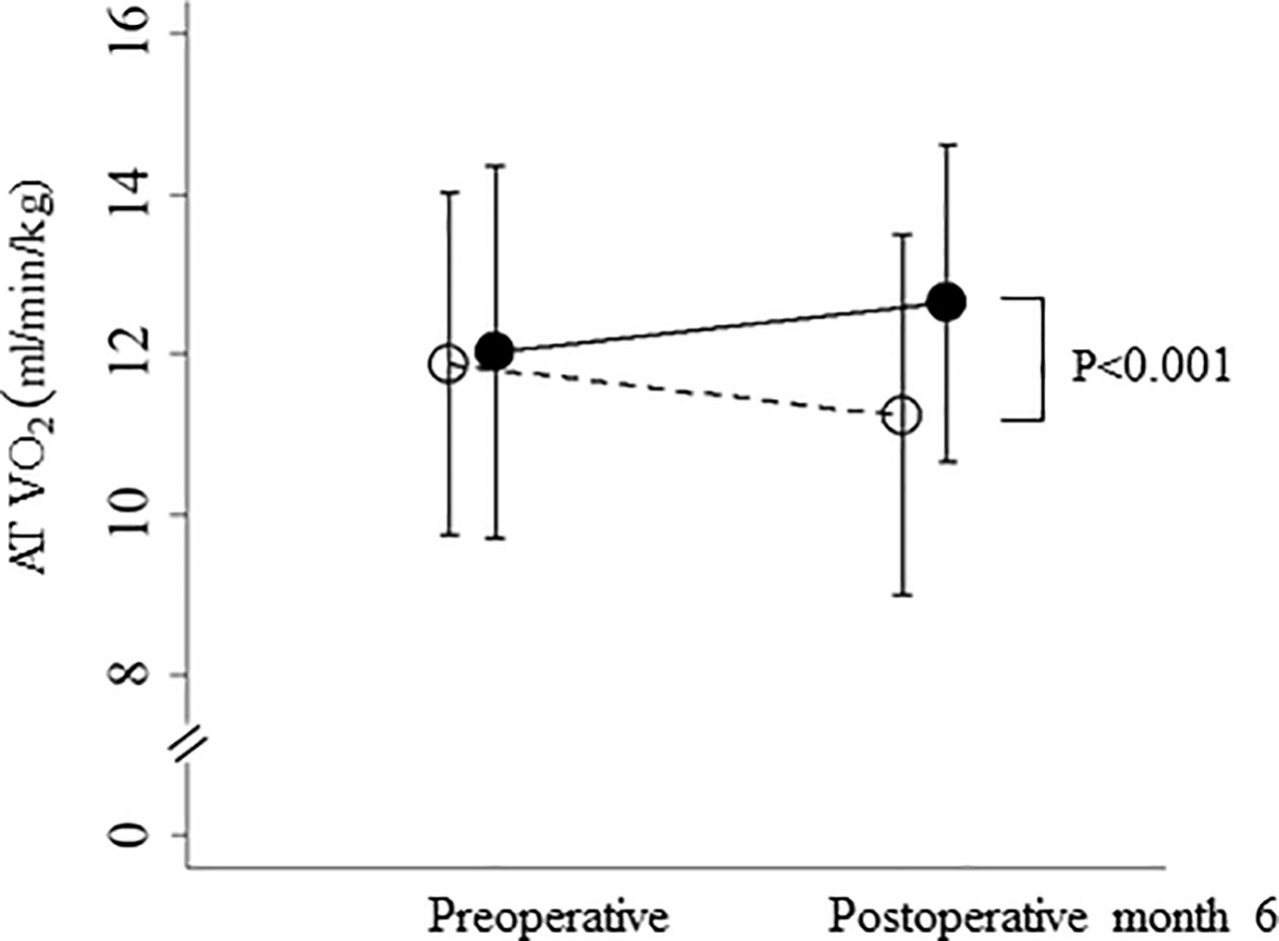
Perioperative changes in AT VO_2_ in the two groups. AT VO_2_ in the maintenance (closed circles) and decrease (open circles) groups after liver resection. Data are shown as the mean ± standard deviation. AT VO_2_: anaerobic threshold as the point between carbon dioxide production and oxygen consumption per unit of time.

In the exercise not-implemented group, mean AT VO_2_ from preoperative to 6 months postoperatively decreased from 11.9 ml/min/kg to 11.2 ml/min/kg, on average, compared with the exercise-implemented group, in which values increased from 12.0 ml/min/kg to 12.6 ml/min/kg, on average, and the difference was significant (p <0.001) (Fig 2).

## Discussion

We examined the relationships between perioperative CPX parameters and long-term survival in 106 patients undergoing hepatectomy. To the best of our knowledge, this retrospective study is the first to investigate the relationship between perioperative cardiopulmonary function related to exercise load and log-term survival in patients undergoing first hepatectomy for HCC. The variables derived from CPX testing included peak VO_2_, which is the maximum oxygen uptake at peak exercise. Previous studies indicated that peak VO_2_ was the most useful predictor of postoperative cardiopulmonary complications in patients undergoing radical esophagectomy with three-field lymphadenectomy [21] and patients undergoing surgical procedures for lung cancer [22–26]. AT is defined as the point during exercise at which oxygen demand outstrips oxygen delivery and metabolism starts to become anaerobic. AT is a measure of the ability of the cardiopulmonary system to deliver adequate oxygen to tissues, and has the advantage of being independent of patient motivation. Reaching AT does not require high levels of physical stress and occurs well before peak VO_2_ [27]. The usefulness of measuring AT has been assessed predominantly in older patients undergoing major surgical procedures, allowing the development of an operative risk grading and treatment protocol [7,8]. An earlier prospective study of preoperative CPX testing in 187 patients undergoing major intra-abdominal surgery demonstrated an association between AT < 11 ml O_2_/kg/min and postoperative cardiovascular mortality [21]. Postoperative complications are associated with reduced fitness, and AT is a prognosticator of postoperative complications [7, 15]; CPX testing has been used in risk stratification. West et al. reported that CPX testing is associated with postoperative morbidity, and a multivariable model including VO_2_ at estimated lactate threshold and gender discriminated those with complications after colonic surgery [28]. Junejo et al. reported that an AT of 9.9 ml O_2_/kg/min predicted inhospital death and subsequent survival in their patients undergoing hepatic resection [29]. In contrast, Dunne et al. reported that when CPX testing is used to delineate perioperative management, a low relative oxygen uptake [VO2 (ml/kg/min)] at does not place patients at significantly higher risk of postoperative complications. This suggested that CPX testing-assessed “poor” fitness should not be used as a barrier to surgical intervention [30]. Although we evaluated preoperative AT VO_2_ value, alone, in our study, preoperative AT VO_2_ was not identified as an independent prognostic predictor for RFS and OS. CPX measurements were performed preoperatively and 6 months postoperatively to evaluate postoperative changes. Therefore, as a future study, we evaluated the rate of change of AT VO_2_ from the preoperative value to 6 months postoperatively as an indicator of prognosis.

Sarcopenia is a strong predictor of outcome after liver resection and orthotopic liver transplantation in HCC patients [31–33]. Therefore, it is important to identify sarcopenia, preoperatively. However, changes in muscle content measured using DEXA in our HCC patients were not associated with long-term survival in this study (Table 4). Although we evaluated preoperative muscle content using DEXA in a multivariable Cox proportional hazards model for RFS and OS, preoperative muscle content was not identified as a significant prognostic indicator (data not shown). However, we did identify perioperative maintenance of AT VO_2_ as an independent prognostic indicator for RFS and OS in our 106 HCC patients (Table 4). Both RFS and OS rates were significantly higher for patients who maintained AT VO_2_ than for patients with decreased AT VO_2_ (Fig 1). Although maintenance of AT VO_2_ correlated with both RFS and OS, the direct mechanism of the association is unclear. Our previous study demonstrated preoperative exercise capacity as an independent prognostic indicator of event-free survival and maintenance of Child–Pugh class in HCC patients with chronic liver injury undergoing hepatectomy [34]. We identified AT VO_2_ < 11.5 ml/min/kg and branched-chain amino acid/tyrosine ratio as independent prognostic indicators of maintenance of Child–Pugh class in 61 HCC patients undergoing hepatectomy. We also found a significant correlation between branched-chain amino acid/tyrosine ratio and AT VO_2_ in HCC patients in our previous study. CPX testing may be useful to predict postoperative recurrence of either HCC or liver dysfunction.

Recently, we found that in patients with HCC and hepatic impairment undergoing liver resection, exercise significantly decreased body mass and fat mass, as well as insulin resistance, 6 months postoperatively [16]. Maintenance of postoperative physical strength and earlier resumption of daily activities could be possible by intensifying perioperative and postoperative exercise. The exercise program we implemented was tailored to each patient who wished to participate. Although mean AT VO_2_ from preoperative to 6 months postoperatively decreased in the exercise not-implemented group, the exercise-implemented group experienced increased AT VO_2,_ on average, 6 months postoperatively (Fig 2). Fifty patients were able to exercise perioperatively in the maintenance group and nine were able in the decrease group; a significantly higher number in the maintenance group (Table 1). Our findings suggest that perioperative exercise is important to maintain postoperative AT VO_2_ after hepatectomy for HCC.

In conclusion, the most significant prognostic factor affecting postoperative survival for chronic liver injury patients with HCC undergoing hepatectomy was maintenance of AT VO_2_ up to 6 months postoperatively, as measured by CPX testing. Our results suggest that exercise involving > 5000 steps per day is necessary for postoperative walking to maintain AT VO_2_, and indicate the importance of introducing perioperative exercise to HCC patients with chronic liver injury.

## Supporting information

S1 File Analysis data as RFS and OS between the two groups. (ZIP)
